# USE OF THE EXERCISE FEATURE OF A HEALTHCARE APP AND CHANGE IN THE PREVALENCE OF LOWER BACK PAIN

**DOI:** 10.1093/geroni/igad104.3686

**Published:** 2023-12-21

**Authors:** Mai Takase, Kishimori Odagami, Hisashi Eguchi, Koji Mori, Masako Itabashi, Sei Kono, Yosuke Shimizu, Katsuya Iijima

**Affiliations:** The University of Tokyo, Tokyo, Tokyo, Japan; University of Occupational and Environmental Health, Japan, Kitakyushu, Fukuoka, Japan; University of Occupational and Environmental Health, Yahata Nishi, Fukuoka, Japan; University of Occupational and Environmental Health, Yahata Nishi, Fukuoka, Japan; Haseko Anesis Corporation, Minato-ku, Tokyo, Japan; Haseko Anesis Corporation, Minato-ku, Tokyo, Japan; Haseko Anesis Corporation, Minato-ku, Tokyo, Japan; The University of Tokyo, Tokyo, Tokyo, Japan

## Abstract

Lower back pain among older adults has been associated with serious conditions, such as depleted cognitive function, and can accordingly limit the degree to which older adults can live independently. Therefore, it is extremely important to engage in exercise and conditioning activities from an early age. We developed a healthcare app with an AI engine that recommends personalized stretch, muscle training, and aerobic exercise programs. These programs were constructed by care prevention exercise experts and are personalized based on the user’s physical balance and the locations of their body pain. This study examined the association between the use of the exercise feature of this app and the change in the prevalence of lower back pain among users. A three-month longitudinal survey with three model companies in Japan (n=730) was conducted from the fall of 2022 to the winter of 2023. Online questionnaire surveys were administered to track the participants’ levels of lower back pain and app logs were retrieved to assess the users’ exercise records. Use of the exercise feature (not used vs. used) was the independent variable and change in the level of lower back pain (none to mild vs. mild to severe) was the dependent variable. A binary logistic regression analysis (n=331, female=34.1%, over 50 years old=43.5%) revealed that the use of the exercise feature led to lower level of back pain (OR = 0.37, 95%CI = 0.29-0.67). This result suggests that the use of this kind of exercise app may help alleviate lower back pain.

